# Comparative Metabolomics Analysis Reveals Key Metabolic Mechanisms and Protein Biomarkers in Alzheimer’s Disease

**DOI:** 10.3389/fphar.2022.904857

**Published:** 2022-05-25

**Authors:** Zhao Dai, Tian Hu, Shijie Su, Jinman Liu, Yinzhong Ma, Yue Zhuo, Shuhuan Fang, Qi Wang, Zhizhun Mo, Huafeng Pan, Jiansong Fang

**Affiliations:** ^1^ Science and Technology Innovation Center, Guangzhou University of Chinese Medicine, Guangzhou, China; ^2^ Department of Rheumatology, First Affiliated Hospital of Guangzhou University of Chinese Medicine, Guangzhou, China; ^3^ Institute of Biomedicine and Biotechnology, Shenzhen Institute of Advanced Technology, Chinese Academy of Sciences, Shenzhen, China; ^4^ Emergency Department, Shenzhen Traditional Chinese Medicine Hospital, Shenzhen, China

**Keywords:** Alzheimer’s disease, metabolome, biomarker, HER2, NDF2

## Abstract

Alzheimer’s disease (AD) is one of the most common progressive neurodegenerative diseases, accompanied by global alterations in metabolic profiles. In the past 10 years, over hundreds of metabolomics studies have been conducted to unravel metabolic changes in AD, which provides insight into the identification of potential biomarkers for diagnosis, treatment, and prognostic assessment. However, since different species may lead to systemic abnormalities in metabolomic profiles, it is urgently needed to perform a comparative metabolomics analysis between AD animal models and human patients. In this study, we integrated 78 metabolic profiles from public literatures, including 11 metabolomics studies in different AD mouse models and 67 metabolomics studies from AD patients. Metabolites and enrichment analysis were further conducted to reveal key metabolic pathways and metabolites in AD. We totally identified 14 key metabolites and 16 pathways that are both differentially significant in AD mouse models and patients. Moreover, we built a metabolite-target network to predict potential protein markers in AD. Finally, we validated HER2 and NDF2 as key protein markers in APP/PS1 mice. Overall, this study provides a comprehensive strategy for AD metabolomics research, contributing to understanding the pathological mechanism of AD.

## 1 Introduction

The prevalence of neurodegenerative disorder dementia is projected to increase from 50 million individuals worldwide in 2010 to 113 million individuals by 2050 (Knopman et al., 2021). Alzheimer’s disease (AD) is the most common cause of dementia leading to cognitive impairment. The core pathological hallmarks of AD is characterized by accumulation of extracellular β-amyloid (Aβ) plaques and intracellular neurofibrillary tangles (NFTs) composed of hyperphosphorylated tau protein (Long and Holtzman, 2019). However, no anti-amyloid or anti-tau small molecule approved by the United States Food and Drug Administration (FDA) currently exist, with many clinical trials for such AD treatments having failed in the past decade (Cummings et al., 2020; Fang et al., 2020). Accumulating evidence have suggested both pathologies of Aβ and tau have synergistic effects but not act independently (Busche and Hyman, 2020). Recently our group have demonstrated that dually targeting the molecular network intersection of amyloid and tau endophenotypes provide the greatest potential in AD drug discovery (Fang et al., 2021).

**FIGURE 1:**
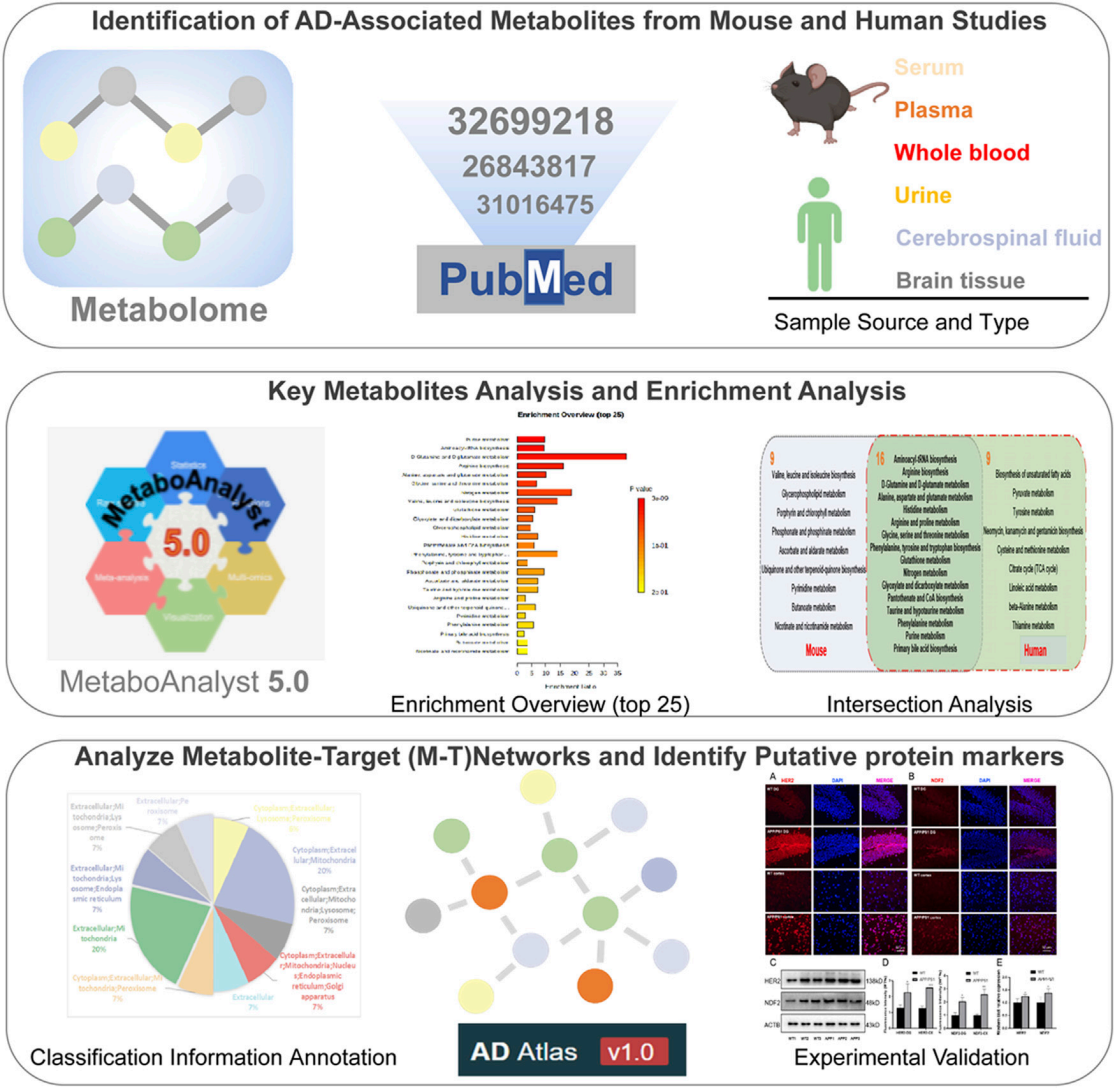
Workflow of the experimental scheme.

Despite significant progress in our understanding of the underlying pathological mechanisms involved in AD, the relationships between systemic abnormalities in metabolism and the pathogenesis of AD are poorly understood. Metabolites are located in downstream of genes and proteins and are most relevant to phenotypic outcomes (Nicholson et al., 2002), allowing more sensitive detection of subtle fluctuations in disease system processes (Bundy et al., 2002). At the same time, it is distinct from the genome and proteome involving different isoforms and is structurally conserved across species. Metabolite detection is an effective means to discover new AD biomarkers (Bundy et al., 2002). Metabolome profiling and positron emission tomography (PET) neuroimaging techniques demonstrate that brain hypoglycemia in AD patients precedes cognitive impairment (Petersen et al., 2000). Proton Magnetic Resonance Spectroscopy (^1^H-MRS) in animal experiments indicates that metabolic changes exist before overt cognitive impairment in early AD (Chen et al., 2012). Large-scale AD multimodal biomarker discovery study suggests that plasma metabolites primary fatty acid amides (PFAMs) have been found increased and associated with Aβ burden in cerebrospinal fluid (CSF) and clinical measures (Min et al., 2019). Integrative metabolomics-genomics approach reveals that increase of circulating adiponectin and metabolite-dependent ABCA1 mRNA expression could be a compensatory effect against Alzheimer’s disease (Horgusluoglu et al., 2021). The analysis of metabolomics has far-reaching significance for in-depth understanding of the pathogenesis of AD and the search for more accurate disease targets. However, based on the complexity of the metabolites, different tissue distributions, different detection techniques, different populations and species can systemic abnormalities in metabolomic profiles. In this study, to compare AD-specific metabolomics alterations between AD animal models and human patients, we integrate multiple metabolic profiles from AD patients (Xie et al., 2021) and mouse models. Significant metabolic pathways and metabolites are identified in AD. Then we further speculate potential targets through constructing and analyzing metabolite-target (M-T) networks, and verify potential protein markers *via* molecular biology experiments (Figure 1).

## 2 Materials and Methods

### 2.1 Identification of Alzheimer’s Disease-Associated Metabolites From Mouse and Human Studies

AD-related mouse brain tissue metabolites were derived from 11 literatures, including four transgenic mouse, which are APPswe/PS1dE9 (APP/PS1), Triple transgenic (3xTg), AβPPswe Tg2576 and 5 familial AD mutations (5xFAD). Detailed animal model information is presented in Supplementary Material. The age of mice covers from 1 to 24 months, and the gender includes male and female. The detection methods involve GC-TOF-MS, ^1^H NMR spectroscopy, MALDI-MSI, etc. All the metabolites collected are significant in data preprocessing with *p* value < 0.05 or adjusted *p* value < 0.05. The detailed information are provided in Table 1.

**TABLE 1:** The summary information of metabolomics studies in AD mouse models.

ID	PMID	Year	Metabolome technique	Animal model	Gender	Age (month)	Sample	Targeted or untargeted	Statistics	Filter criteria
1	32699218	2020	UPLC-QTQF, LC-MS, MRM HILIC–LC–MS/MS, HILIC–LC–QTOF, GC–MS	APP/PS1	Male	6, 12, 24	Brain, spleen	Both	t test	*p* < *0.05*
2	33197957	2020	UHPLC-MRM-MS	3xTg	Male	2, 6	Hippocampus	Targeted	Student’s t test	*p* < *0.05*
3	26843817	2015	UPLC/MS	APP/PS1	Male	7	Brains	Unknown	Student’s t test	*p* < 0.05, VIP < 1.5
4	31016475	2019	GC-TOF-MS	APP/PS1	Both	4.5	Brain (cortex, hippocampus, midbrain, cerebellum), plasma	Unknown	Two-tailed independent t test	*p* < 0.05
5	26827653	2015	Quantitative mass spectrometry	APP/PS1	Male	6, 8, 10, 12, 18	Brain, Plasma	Targeted	Nonparametric 1-way analysis of variance analysis (Kruskal-Wallis)	*p* < 0.05
6	32033569	2020	LC-MS	5xFAD	Male	6, 8, 12	Hippocampal	Untargeted	Independent t test	FDR adjusted *p*-value (q-value) < 0.05
7	29107091	2017	^1^H NMR spectroscopy	APP/PS1	Male	1, 5, 10	Brain	Unknown	Student’s t test with Bonferroni adjustment	Bonferroni adjusted *p* value < 0.05
8	28411106	2017	MALDI-MSI	3xTg	Male	unknown	Brain	Untargeted	t test	*p* < 0.01
9	24145382	2014	^1^H NMR spectroscopy	Tg2576	Male	1, 3, 6, 11	Brain (frontal cortex, rhinal cortex, hippocampus, midbrain, cerebellum)	Unknown	CV-ANOVA	Q2 > 0.5, *p*-value < 0.05, response permutation test, *p*-values of t-tests <0.05
10	25281826	2014	GC–MS UPLC–MS	APP/PS1	Both	6	Brain (cortex and cerebellum)	Unknown	t-test with Bonferroni correction	VIP < 1.5, *p* < 0.05
11	25459942	2014	QTOF-MS	APP/PS1	Both	6	Brain (hippocampus, cortex, cerebellum, olfactory bulbs)	Untargeted	t-test with Bonferroni correction	VIP < 1.5, *p* < 0.05

AD-related human metabolites were collected from one recent systematic review (Xie et al., 2021), which summarized 67 AD or mild cognitive impairment (MCI) patient metabolic profiling studies. Among the 67 studies, the number of AD cases ranged from 7 to 1,356, the number of MCI cases ranged from 10 to 356, and the number of healthy controls ranged from 7 to 23,882. The biosample sources included serum, plasma, brain tissue, cerebrospinal fluid, saliva, and urine. We then analyzed the frequency of differential metabolites in these studies. The detailed information is presented in Supplementary Table S1.

### 2.2 Pathway Enrichment Analysis

We used the online analysis platform MetaboAnalyst 5.0 (https://www.metaboanalyst.ca) to analyze 27 differential metabolites in AD mice and 60 human differential metabolites pathway enrichment based on extraction from the original study. The website contains statistical analysis, enrichment analysis, pathway analysis, functional analysis, etc. Pathway enrichment is classified and compared according to different biological samples (mouse or human). Brain tissue specificity data for each gene comes from AlzGPS (https://alzgps.lerner.ccf.org). Subcellular localization sourced from COMPARTMENTS (https://compartments.jensenlab.org/).

### 2.3 Construction of Metabolite-Centric Subnetworks Using Alzheimer’s Disease Atlas

Metabolite related genes information comes from AD atlas (https://www.adatlas.org/). The AD Atlas is a dataset resource for studying AD-related biomarkers and AD-related endophenotypes based on a multi-omics background. It provides an interactive network of phenotypes, genes, proteins or metabolites. In this work, 14 common metabolites between AD mouse and human studies were input into AD Atlas to generate a metabolite-centric subnetwork. We used genome-wide as significance threshold, brain cortex to filter coregulation links. Five interaction types were covered in network, including coexpression, coregulation, genetic association, metabolic association, and partial correlation. Furthermore, the topology analysis in cytoHubba plug was used to measure important nodes in the network graph.

### 2.4 Experimental Validation

#### 2.4.1 APPswe/PS1dE9 Animal Models

APP/PS1 male mouse and wild type (WT) mouse were purchased from Guangzhou Dean Gene Technology Co., Ltd. 8.5 and 17 month-old APP/PS1 mice (*n* = 10), and matched WT mice (*n* = 10) were selected for experiments respectively. All experimental procedures follow the requirements of the Ethical Review Regulations for Laboratory Animals of Guangzhou University of Chinese Medicine. Mice were housed in single cages in an air-conditioned room with a relative humidity of 40%–70% and a temperature of 20–26°C with a light-dark cycle for 12 h. Mice have free access to food and water.

#### 2.4.2 Western Blot Analysis

We selected mouse brain cortex for western blot analysis. Add 10 μl of RIPA lysate containing phosphatase inhibitor and PMSF to each 1 mg of tissue. After treatment with a cell sonicator or tissue homogenizer, centrifuge to take the supernatant, add 1/4 of the total volume of loading buffer, and boil at 100°C for 5 min to completely denature the protein. The protein lysates were separated in equal volume on 10% Precast Protein Gels and transferred to an 0.22 μm Immobilon-PSQ polyvinylidene difluoride membrane (PVDF, Millipore, cat. #ISEQ00010). The following antibodies were used: Neuronal differentiation 2 (NDF2) Antibody (1:1000, Affnity Biosciences, cat. #AF0635); Human EGF Receptor (HER2/ErbB2) Antibody (1:1000, Affnity Biosciences, cat. #AF7681); beta Actin Antibody (1:3000, Affnity Biosciences, cat. #AF7018). At the end of the immunoreaction, Immobilon Western Chemilum HRP Substrate (Millipore, cat. #WBKLS0100) was prepared in a 1:1 ratio, and the western blot was recorded by a versatile gel imager ChemiDocXRS+ (Bio-RAD, United States).

### 2.4.3 Immunofluorescence

The 3 μm coronal brain slices were blocked in 5% BSA at 37°C for 1 h, then incubated with primary antibodies at 4°C overnight. The next day, the slices were washed three times in PBS for 5 min each and incubated with corresponding secondary antibodies at 37°C for 2 h. The slices were stained with DAPI nuclear staining (Cell Signaling Technology, cat. #4083) for 1 min. Use the same antibody as for western blot.

### 2.5 Network Visualization and Statistical Analysis

Statistical analysis of this study was done by SPSS, GraphPad Prism 8 (v8.0.2, https://www.graphpad.com/). The mean ± standard deviation was used for statistical description, and the independent sample T test was used to compare with the control group, *p < 0.05, **p < 0.01, ***p < 0.001. The network was constructed using Cytoscape (v3.2.1, http://www.cytoscape.org/), GraphPad Prism 8, and Microsoft Office 2019.

## 3. Results

### 3.1 Collection of Differential Metabolites in Mouse and Human Metabolomics

**FIGURE 2:**
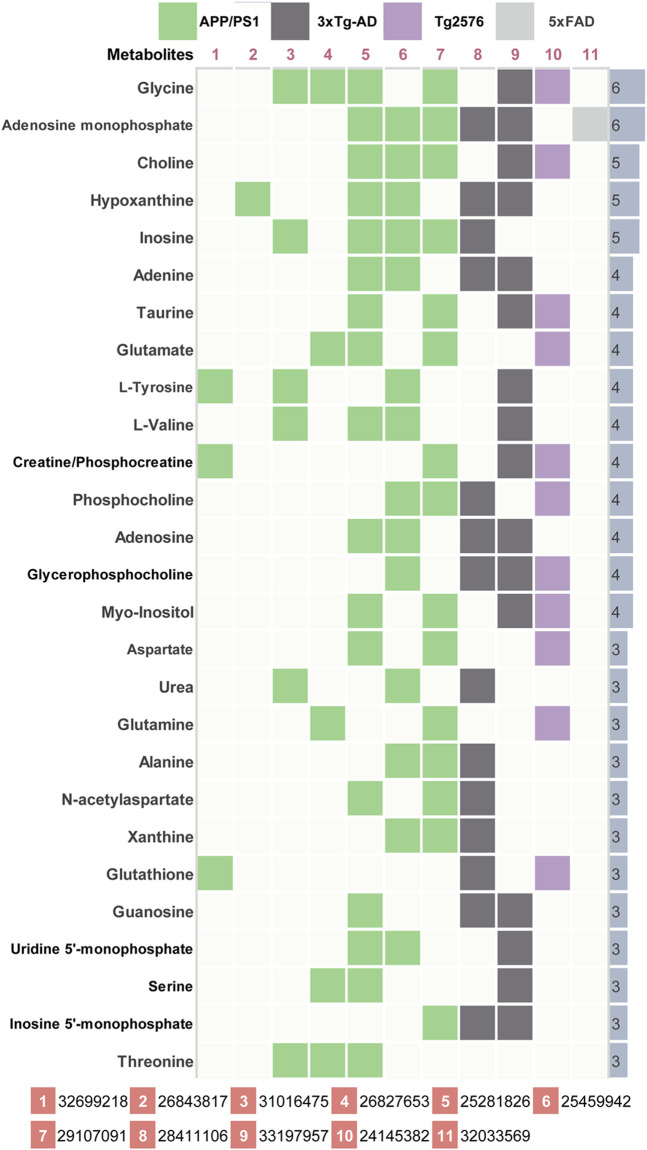
Key differential metabolites in AD model mice. 11 previous metabolomics studies (PMID number) using AD mouse brain tissue were included. The key metabolites were listed together with their significant frequency in these studies.

AD-related differential metabolites were collected from 11 previous metabolomics studies using AD mouse brain tissue. Seven of them are APP/PS1 transgenic mouse models, two are 3xTg transgenic mouse models, and the remaining two are Tg2576 and 5xFAD transgenic mouse model, respectively. A total of 408 differential metabolites were identified in the 11 literatures. After analysis and sorting, 27 key metabolites in mouse brain tissue were obtained by taking the frequency of three or more reports (Figure 2). Among the 11 studies, the study (González-Domínguez et al., 2014) has the highest overlap (*n* = 17) compared with the 27 differential metabolites. In addition, we observe that both of two metabolites (glycine, adenosine monophosphate) were detected in six studies. *In vitro* studies show that glycine increases viability and counteracts deleterious responses to LPS/IFN-γ-induced apoptosis in BV-2 microglia (Egger et al., 2020). *In vivo* studies have demonstrated that glycine exerts neuroprotective effects by mediating inactivation of the JNK signaling pathway, and it can reverse D-galactose-induced oxidative stress, neuroinflammation, synaptic dysfunction, and memory impairment (Rahat et al., 2020). Likewise, cyclic adenosine monophosphate (cAMP) as an endogenous modulator of the amyloid-beta precursor protein metabolism (Canepa et al., 2013). cAMP controls amyloid precursor protein (APP) translation and Aβ levels, regulates long-term potentiation (LTP) and ameliorates memory in healthy and diseased brain (Ricciarelli et al., 2014). These findings confirm the veracity and accuracy of our research.

**FIGURE 3:**
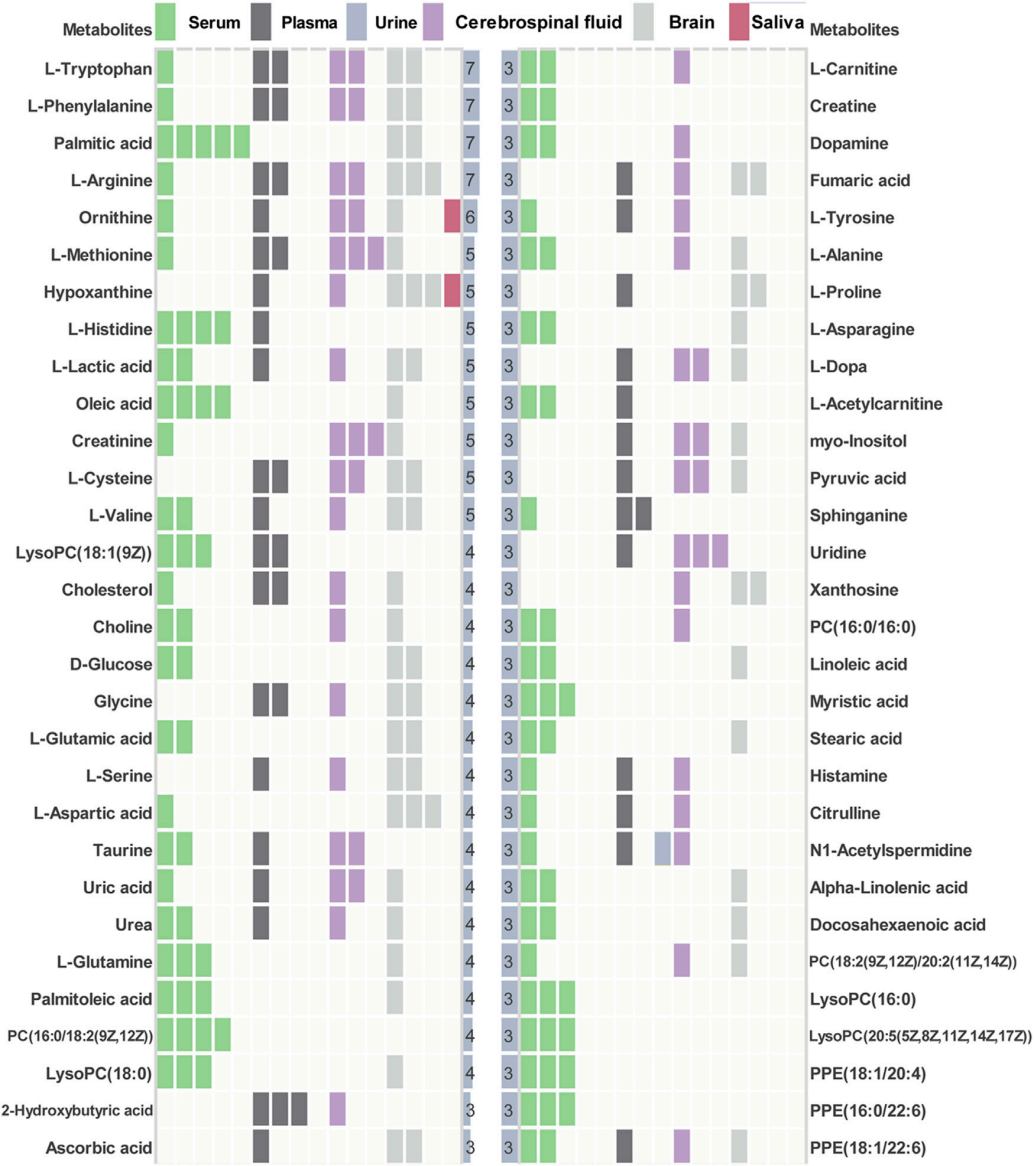
Key differential metabolites in AD patients. Key metabolites with significant frequencies greater than three are listed and colored to distinguish different tissue types.

Among 67 human metabolomics studies, there were 830 altered metabolites in AD patients compared to healthy individuals, of which 60 metabolites were reported three or more times (Figure 3). These 60 metabolites cover 37 literatures. Four metabolites (L-tryptophan, L-phenylalanine, palmitic acid, L-arginine) were detected significant in 7 studies. *In vitro* studies demonstrated that extra-cellular palmitic acid coupled with G protein-coupled receptor 40 (GPR40) induces the expression of APP and BACE1 to facilitate Aβ production *via* the Akt-mTOR-HIF-1α and Akt-NF-κB pathways in SK-N-MC cells (Kim et al., 2017). In addition, studies have shown that palmitic acid abolished the migration and phagocytic activity of microglia in response to interferon-γ, thereby aggravating cellular inflammatory damage (Yanguas-Casas et al., 2018). A mouse model of CVN-AD, in which immune-mediated nitric oxide is reduced to mimic human levels, found areas of hippocampal neuronal death associated with the presence of immunosuppressive CD11c (+) microglia and extracellular arginase, resulting in decreased arginine catabolism and total levels of brain arginine (Kan et al., 2015). Moreover, a study of male APPswe/PS1ΔE9 transgenic (Tg) showed significantly altered plasma arginine metabolism profiles in 7-month Tg mice prior to major behavioral impairment (Bergin et al., 2018). These studies provide cogent evidence on the importance of high repetition rate metabolites in AD.

### 3.2 Key Metabolites Analysis and Enrichment Analysis

**FIGURE 4:**
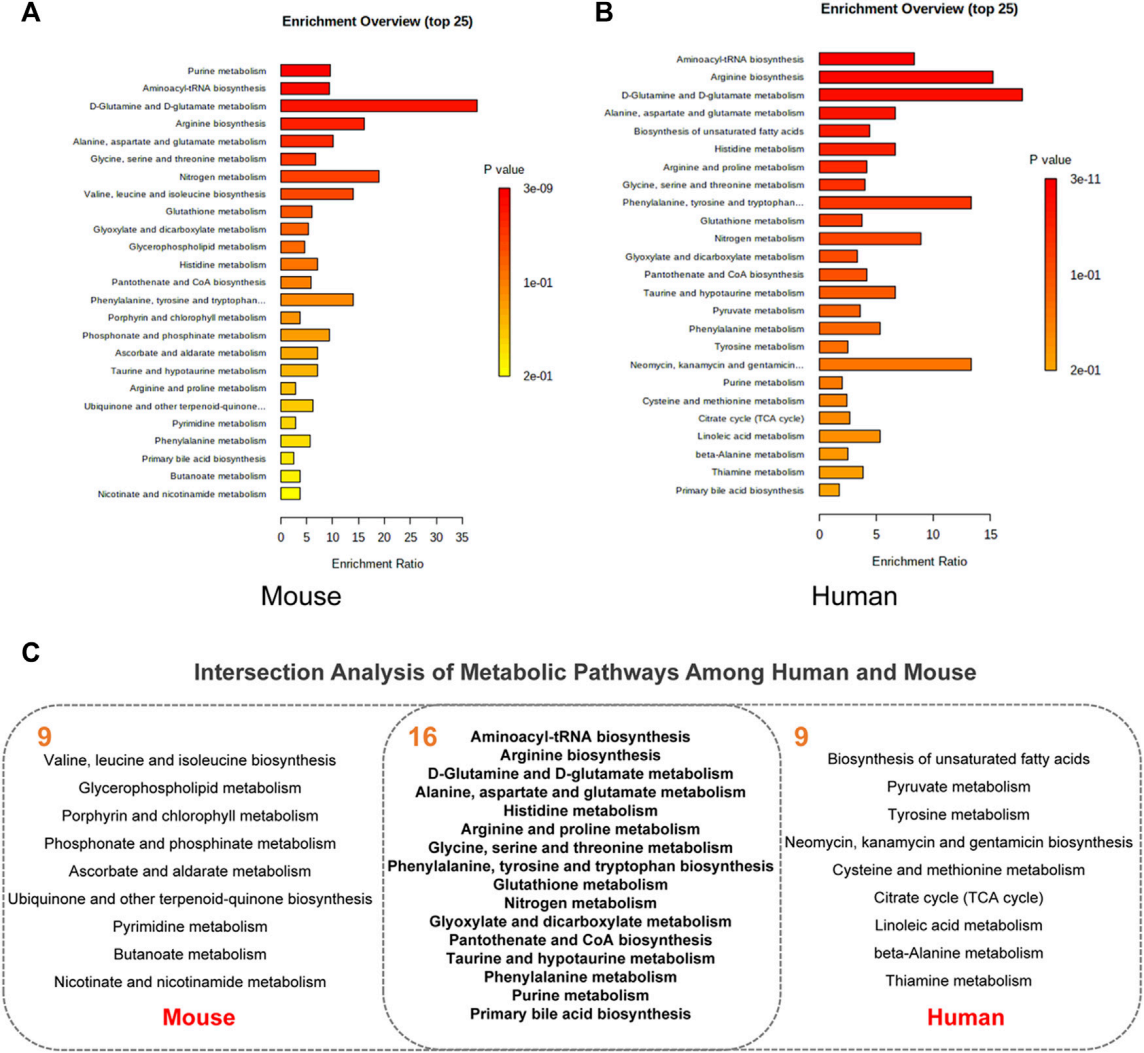
Pathway enrichment analysis. **(A)** The top 25 pathway in AD animal models. **(B)** The top 25 pathway in AD patients. **(C)** Intersection analysis of metabolic pathways among human and mouse.

Pathway enrichment analysis was performed for key differential metabolites and top 25 significant pathways were kept for both types of metabolites. As shown in Figure 4A, analysis of 27 differential metabolites in AD mice revealed that 33 pathways exhibited alterations in the AD-related mouse brain tissue. The top 25 pathways were mainly related to D-glutamine and D-glutamate metabolism (Enrichment Ratio = 37.74, *p* < 0.001), nitrogen metabolism (Enrichment Ratio = 18.87, *p* < 0.01), arginine biosynthesis (Enrichment Ratio = 16.13 *p* < 0.001) and valine, leucine, and isoleucine biosynthesis (Enrichment Ratio = 14.08, *p* < 0.01). As for 60 key differential metabolites in AD patients (Figure 4B), there are 50 pathways that exhibited alterations in the AD patients. The top 25 pathways were mainly related to D-glutamine and D-glutamate metabolism (Enrichment Ratio = 17.78, *p* < 0.001), arginine biosynthesis (Enrichment Ratio = 15.27, *p* < 0.001), phenylalanine, tyrosine and tryptophan biosynthesis (Enrichment Ratio = 13.33, *p* < 0.01), and aminoacyl-tRNA biosynthesis (Enrichment Ratio = 8.33, *p* < 0.001). Enrichment Ratio is computed by hits/expected, where hits = observed hits; expected = expected hits. Details of pathway enrichment are presented in Supplementary Tables S2, S3.

### 3.3 Intersection Analysis of Metabolic Pathways Between Human and Alzheimer’s Disease Mouse

We further aimed to examine the common regulated metabolic pathways between AD patients and AD mice. Figure 4C presents 16 overlapping pathways between them. Most of these pathways have high enrichment ratio and low *p* values, such as D-glutamine and D-glutamate metabolism, arginine biosynthesis, alanine, aspartate, and glutamate metabolism. Glutamate is the most common excitatory transmitter in the mammalian central nervous system and plays an important role in normal brain function and brain development. Low synaptic clearance and excitotoxicity of glutamate resulting from altered glutamatergic neurotransmission are thought to play critical roles in AD progression (Acosta et al., 2017; Nuzzo et al., 2021). A recent study has demonstrated that subjects with MCI show paradoxical elevations in glutamatergic presynaptic bolton density, which are then depleted and decreased as the disease progresses (Bell et al., 2007). Recently, substantial evidence implicates that altered L-arginine metabolism is involved in AD pathogenesis (Griffin and Bradshaw, 2017; Meloni et al., 2020). Arginase converts L-arginine to L-ornithine, which is further metabolized by ornithine decarboxylase (ODC) to the polyamine putrescine (Wu and Morris, 1998). Polyamines promote the aggregation of Aβ oligomers and reduce their toxicity by reducing the hydrophobic surface and increasing the size of the oligomers (Luo et al., 2013) and are essential for the maintenance of normal cellular function. In a study on APP/PS1 mice, the content of L-arginine continued to increase with age. Amyloid plaques were distributed widely across the brain at 17 months of age, the content of L-arginine in this time was significantly higher than that of the normal group (Vemula et al., 2020). Collectively, accumulating evidence has supported that these metabolic pathways play important roles in both AD patients and animal models.

### 3.4 Analyze Metabolite-Target Networks and Identify Putative Protein Markers in Alzheimer’s Disease

**FIGURE 5:**
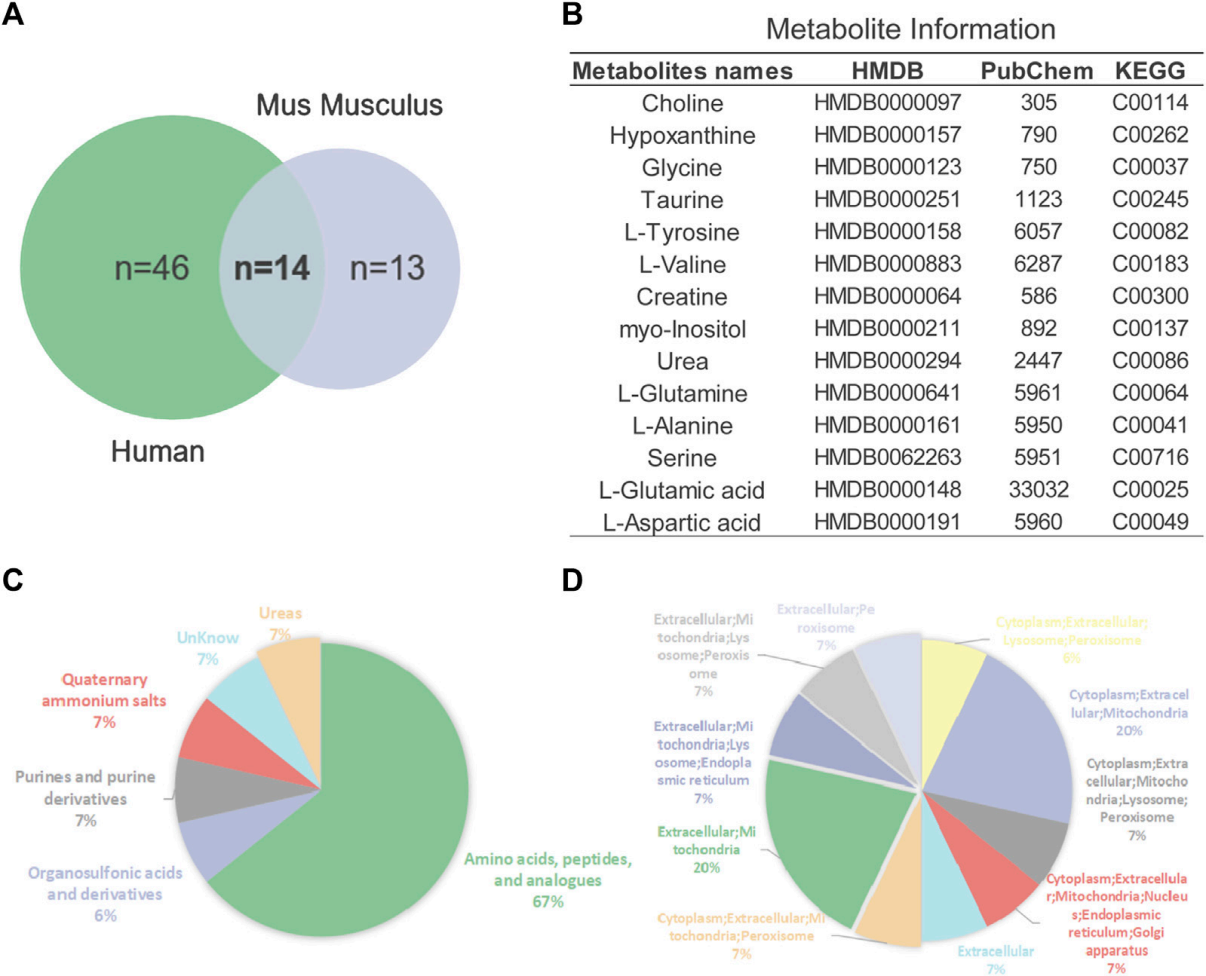
Analysis of key metabolites. **(A)** Venn diagrams of animal and human metabolites. **(B)** The 14 key metabolites information. **(C)** Annotation and statistics of chemical classification information of metabolites. **(D)** Subcellular localization analysis of metabolites.

In addition to 16 overlapping enriched pathways, we also found 14 key metabolites overlap between AD mouse models and patients (Figure 5A). Their detailed information is presented in Figure 5B. Annotation and statistics were performed on the chemical classification and attribution information of metabolites (Figure 5C). Metabolites in different categories were displayed in different color blocks. Nine metabolites belong to amino acids, peptides, and analogues (creatine, glycine, alanine, aspartate, glutamate, glutamine, serine, valine, tyrosine), metabolite taurine belongs to organ sulfonic acids and derivatives, metabolite hypoxanthine belongs to purines and purine derivatives, metabolite choline belongs to quaternary ammonium salts, metabolite urea belongs to ureas and myo-Inositol unclear attribution. Subcellular localization analysis of metabolites was performed based on the information included in the Human Metabolome Database (HMDB) (Figure 5D). All metabolites are distributed extracellularly (*n* = 14) and most metabolites are distributed in mitochondria (*n* = 11), in contrast, only choline is distributed in the nucleus and golgi apparatus, which is the most widely subcellularly localized metabolite (cytoplasm, extracellular, mitochondria, nucleus, endoplasmic reticulum, golgi apparatus). Choline, a B vitamin nutrient found in common foods, is important for various cellular functions. Experiments demonstrate that lifelong choline supplementation significantly reduces amyloid-beta plaque burden and improves spatial memory in APP/PS1 mice (Velazquez et al., 2019).

**FIGURE 6:**
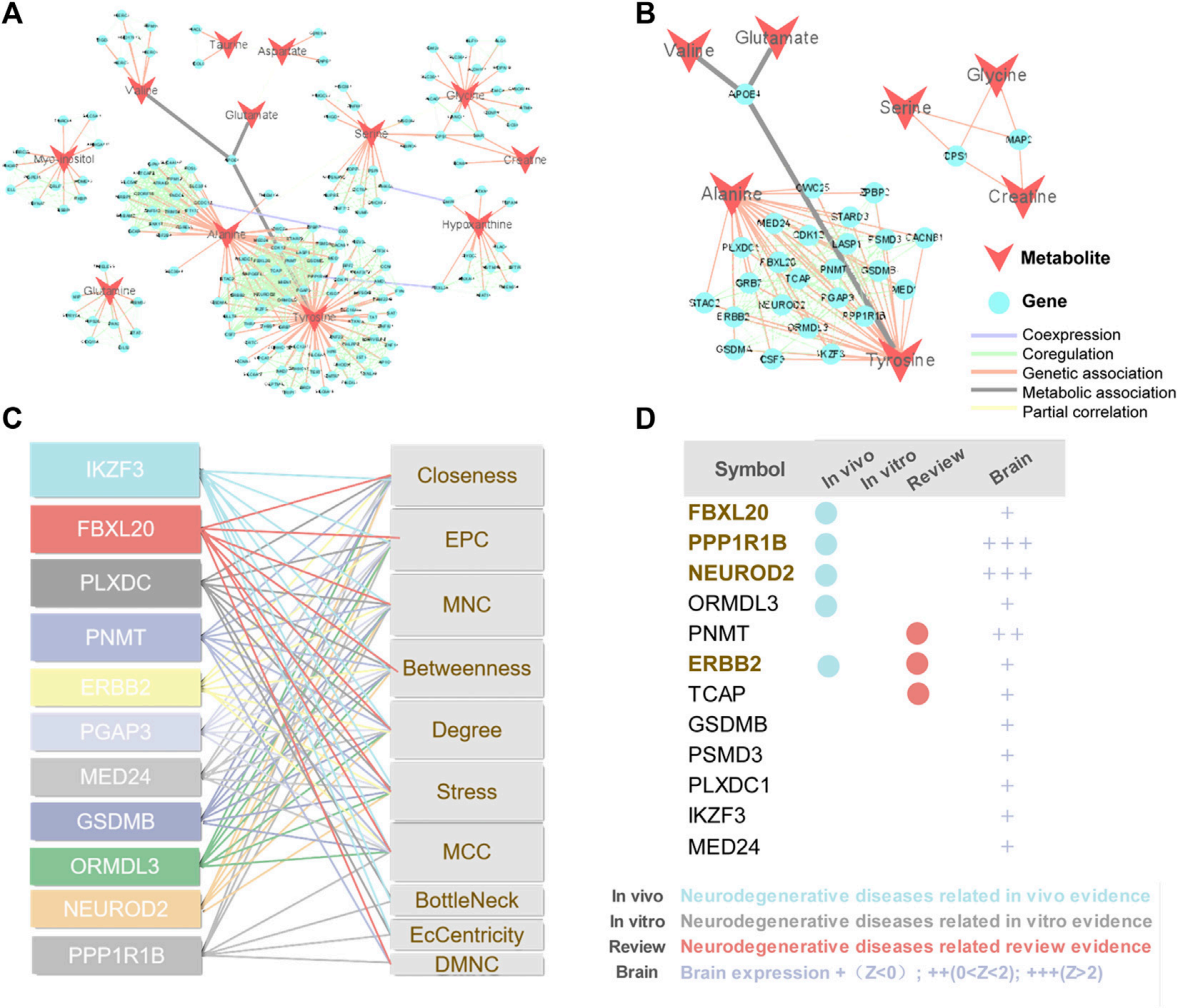
Construction of M-T networks and identification of putative protein markers in AD. **(A)** Metabolite target (M-T) total network. The metabolites and proteins are labeled with different node shapes and node colors, and the interaction relationships are labeled with different line thicknesses and line colors. **(B)** Metabolite target (M-T) sub-network. **(C)** Topological analysis of targets in M-T network. **(D)** Literature evidence and brain expression of targets. Brain expression data (z score) for each gene comes from AlzGPS (https://alzgps.lerner.ccf.org).

Metabolites are downstream of the genome, transcriptome, and proteome, whereas proteins are the ultimate carriers of functions in an organism. In biological systems, metabolite-protein interactions control a variety of cellular processes, therefore, we used AD Atlas to construct M-T network, in order to find potential AD markers associated with metabolites (Figure 6A). The M-T networks include 12 metabolites except choline and urea. We retained proteins with linkages to at least two metabolites, resulting in a sub-network diagram (Figure 6B). Ten topology analysis methods, including Closeness, EPC, MNC, Betweenness Degree, Stress, MCC, BottleNeck, EcCentricity and DMNC are used to measure the true efficiency of nodes in the sub-network to evaluate their importance (Figure 6C). Topological analysis has sorted out the 11 most important proteins in the network diagram. Combined with the expression levels in the brain (Zhou et al., 2021) and the supporting literature evidence related to neurodegenerative diseases *in vivo* and *in vitro*, we screened out FBXL20, PPP1R1B, NEUROD2 (NDF2), and ERBB2 (HER2) for follow-up molecular biology experiments (Figure 6D).

### 3.5 Experimental Validation of Human EGF Receptor and Neuronal Differentiation 2 as Putative Protein Markers in APP/PS1 Models

**FIGURE 7:**
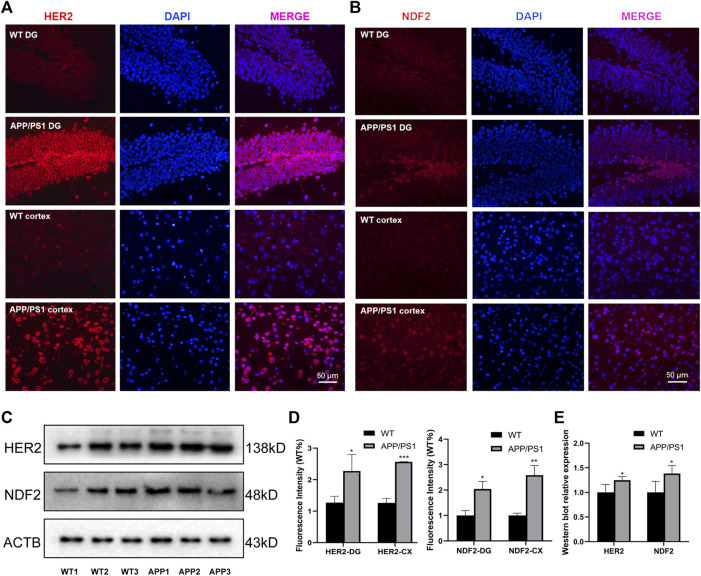
Experimental validation of HER2 and NDF2. **(A)** Immunofluorescence expression of HER2 (8.5 months). **(B)** Immunofluorescence expression of NDF2 (8.5 months). **(C)** Western blot results of NDF2 and HER2 (17 months). **(D,E)** Statistical analysis of immunofluorescence and western blot. *p < 0.05, **p < 0.01, ***p < 0.001.

We selected APP/PS1 mice in two different months of age to verify the four proteins selected in the above analysis, of which FBXL20 and PPP1R1B had no significant difference between WT and APP/PS1 mice. 8.5-month-old APP/PS1 mice exhibited abundant Aβ accumulation in the hippocampus and cortex (Jankowsky et al., 2004), while 17-month-old APP/PS1 mice exhibited learning impairment (Savonenko et al., 2005). Figure 7A shows the immunofluorescence expression of the protein HER2 of 8.5-month-old APP/PS1 mice, the results show that the expression in the hippocampal dentate gyrus area and cortical area of APP/PS1 is significantly higher than that in WT (*p* < 0.05, *p* < 0.001). Figure 7B shows the immunofluorescence expression of the protein NDF2, and the results show that the expression in the hippocampal dentate gyrus area and cortical area of APP/PS1 is significantly higher than that in WT (*p* < 0.05, *p* < 0.01). Moreover, Figure 7C shows the western blot expression of the proteins NDF2 and HER2 of 17-month-old APP/PS1 mice. The results showed that the expressions of NDF2 in APP/PS1 mice is significantly higher than that in WT (*p* < 0.05) and HER2 in the brain tissue of APP/PS1 mice is significantly higher than that in WT (*p* < 0.05).

## 4 Discussion and Conclusion

Metabolites drive cells to perform basic functions including signal transduction, energy production and storage. As the biochemical products of the cellular process, metabolites reflect alterations in biochemical pathways related to the pathogenesis of AD. Thus, studying metabolites has profound implications in the identification of potential biomarkers for diagnosis, treatment, and prognostic assessment.

In this work, we perform a comparative metabolomics analysis to offer a global view of metabolic changes between AD animal models and AD patients. We integrate multiple metabolic profiles from public data source, and conduct metabolites and metabolic pathway overlap analysis. The key metabolites and enriched pathways are identified, which are both differentially significant in AD mouse models and patients. Through construction of M-T network, we finally predicted and experimentally validated HER2 and NDF2 as key protein markers in APP/PS1 mice.

HER2, commonly known as Erb-B2 Receptor Tyrosine het Kinase 2 (ErbB2), is a member of the epidermal growth factor receptor (EGFR) family, including four members EGFR (ErbB1, HER1), ErbB2 (HER2), ErbB3 (HER3) and ErbB4 (HER4). In peripheral nerve regeneration after trauma, HER2 regulates Schwann cell proliferation, yet its high expression drives cancer development and progression in certain tissue types. The important reason of HER2 receptor leading to carcinogenesis is ligand-independent activation, which activates downstream signals. Neuregulin-1 (Nrg1) is a member of the active epithelial growth factor (EGF) family (Patricia et al., 2005), which binds to ErbB receptors, NRG1 is a ligand of ErbB3 or ErbB4, and is more likely to heterodimerizes with ErbB2 after binding to ErbB3 or ErbB4 aggregates. Nrg1 mediated intercellular and intracellular communication regulates multiple biological processes involved in nervous system development by binding to ErbB receptors, and they play key roles in regulating nervous system development and regeneration. NRG1 signaling in microglia stimulates microglial proliferation, chemotaxis, and survival through the ErbB2 receptor, as well as interleukin-1β release *in vitro* (Calvo et al., 2010). Changes in ErbB expression appear to play an important role in nerve injury and subsequent nerve regeneration (Calvo et al., 2010; Kanzaki et al., 2013; Ronchi et al., 2013). NRG accelerates Schwann cell migration through ErbB2/3-dependent FAK pathway, thereby promoting nerve regeneration (Hung-Ming et al., 2013). NRG1 activates heteromeric ErbB2 and ErbB3 receptors on Schwann cells and plays an important role in Schwann cell development and myelination (Garratt et al., 2000; Lemke, 2006). Plasma soluble neuregulin-1 levels were significantly elevated in mild AD patients and moderate AD patients compared with healthy controls (Shin et al., 2016). cDNA microarray technology was used to study the changes of gene expression profiles in the cerebral cortex of Balb/c mice injected with Aβ fragment 25–35 into the ventricle, and it was found that ErbB2 gene expression was upregulated (Kong et al., 2010).

NDF2, commonly referred to as Neurod2, is a neurogenesis marker in the hippocampal dentate gyrus and is thought to play a role in the determination and maintenance of neuronal cell fate. The findings suggest that alterations in Neurod2 can lead to neurodevelopmental disorders, including intellectual disability and autism spectrum disorder (Runge et al., 2021). Higher levels of RE1-silencing transcription factor (REST) were detected in the hippocampus of Neurod2 knockout mice. REST inhibits neuronal differentiation, and the neuronal differentiation program must be activated for progenitor cells to become neurons (Ravanpay et al., 2010). In the APP/PS1 mouse model, targeted expression of Neurod1 in hippocampal progenitor cells significantly reduced the defect in dendritic spine density in new hippocampal neurons, and the highly connected new neurons were able to restore spatial memory in these diseased mice (Kevin et al., 2014). Reduced neurogenesis and synaptic plasticity, thought to be associated with age-related cognitive decline (Bettio et al., 2017). Memory deficits, a typical feature of AD, develop decades before they are detected as mild cognitive impairment. New neurons and glial cells in the adult hippocampus are added to the granular cell layer of the dentate gyrus committed to learning and memory. Conversely, defects in neurogenesis with aging, neuroinflammation, oxidative damage, mitochondrial dysfunction, etc., may impair hippocampal function and gradually lead to memory loss. The enhanced neurogenesis in our experiments may be a compensatory response, representing an endogenous brain repair mechanism.

There are two novel findings in our study. First, our comparative metabolomics analysis integrating multiple data sources revealed the metabolite similarities and differences between AD patients and animal models, confirming that the existing AD animal models have good commonality with AD patients at the metabolite level. Secondly, two potential protein markers (HER2 and NDF2) were predicted and verified in APP/PS1 mouse model, which provided some new directions and explorations for AD research. However, several limitations of the presented study should be recognized. First, our data was derived from the differential metabolites listed in the literature and lack a standardized analytical workflow for sample acquisition, processing and preparation, instrumental analysis, and data analysis. Second, the included data was mostly cross-sectional studies, while the early diagnostic and prognostic potential of metabolite biomarkers should be further assessed in high-quality, large-scale longitudinal cohort studies. Third, due to insufficient data on patient brain tissue, 67 metabolomics studies from AD patients included multiple tissue sources, compared with only brain tissue source in AD mouse models. This might have some impact on the results.

Generally, metabolomics is an exciting and growing field of research, and its study has illuminated its ability to expand from biomarker discovery to understanding the mechanisms behind phenotypes. Our study may bring some directions and implications for AD metabolomics research, providing a comprehensive understanding of HER2 and NDF2.

## Data Availability

The original contributions presented in the study are included in the article/Supplementary Material, further inquiries can be directed to the corresponding authors.
