# Supplementary material for: Comparative Metabolomics Analysis Reveals Key Metabolic Mechanisms and Protein Biomarkers in Alzheimer’s Disease
Source: Front Pharmacol. 2022 May 25;13:904857. doi: 10.3389/fphar.2022.904857 (PMC9174950; doi:10.3389/fphar.2022.904857)
Supplement: Supplementary file 1 [file DataSheet1.docx]

**Supplementary Table 1 The summary information of metabolomics studies in AD patients**

| **PMID** | **Study Type** | **Number of Subjects** | **Sample Source** | **Sample Type** | **Platforms** |
| --- | --- | --- | --- | --- | --- |
| 29370177 | Case-control study | 15 AD 15 ASYMAD 14 CN | BLSA | Brain | FIA-MS/MS；HPLC-MS/MS |
| 29375291 | Case-control study | 37 AD 46 CN | Florida Hospital’s NPH program | Serum | GC-GC-TOFMS |
| 25585166 | Case-control study | 36 AD 48 MCI 40 CN | Dementia Case Register (DCR) at King’s College London and the EU funded AddNeuroMed study | Plasma | UPLC-MS |
| 28951883 | Case-control study | 127 AD 121 CN | Amsterdam Dementia Cohort | Plasma | UPLC-MS;GC-MS |
| 26957286 | Case-control study | 9 AD 9 CN | New Zealand Neurological Foundation HBB | Brain | GC--MS |
| 26717242 | Case-control study | 21 AD 19 CN | Banner Sun Health Research Institute | Brain | UPLC-HILIC-MS; LC-MS |
| 27069549 | Case-control study | 14 AD 15 CN | Newcastle Brain Tissue Resource | Brain | GC--MS |
| 26806385 | Nested case control study | 193 AD 199 CN | BLSA and AGES-RS | Serum | FIA-MS/MS;HPLC-MS/MS |
| 26825096 | Case-control study | 75 AD 17 MCI 45 CN | Neurological Service of Hospital Juan Ramón Jiménez | Serum | UPLC-MS |
| 27255810 | Case-control study | 35 AD 38 MCI 34 CN | Memory clinic of the Santa Lucia Foundation (Rome, Italy) | Plasma | GC-MS; LC-MS |
| 25408209 | Case-control study | 50 AD-Like 50 CN | Unknown | CSF | FIA-MS/MS ; HPLC-MS/MS |
| 26744734 | Case-control study | 43 AD 33 MCI 35 CN | The memory clinics at the Department of Psychiatry of Innsbruck Medical University and Hall in Tirol State Hospital | Plasma | AbsoluteIDQ p180 Kit (Biocrates Life Sciences) |
| 25281130 | Case-control study | 30 AD 30 CN | Neurologic service of Hospital Juan Ramón Jiménez (Huelva, Spain) | Serum | FIA-APPI-QTOF MS |
| 26405996 | Case-control study | 33 AD 31 CN | Centre of Harbin Elderly Care Service in Heilongjiang Province of northern China. | Serum | GC-MS |
| 25311278 | Case-control study | 16 AD 4 CN | Institute of Neuropathology HUB-ICO-IDIBELL Biobank | Brain | LC-MS |
| 24473279 | Case-control study | 19 AD 17 CN | Neurologic Service of Hospital Juan Ramón Jiménez (all residents in the province of Huelva) | Serum | UPLC-ESI-QTOF MS; UPLC-ICP-MS |
| 25136972 | Case-control study | 42 AD 14 MCI 37 CN | Neurological Service of Hospital Juan Ram´on Jim´enez | Serum | CE-ESI-TOF-MS |
| 25515716 | Case-control study | 20 AD 20 MCI 20 CN | Hengjing Affiliated Hospital, China Medical University | Plasma | UPLC-MS |
| 25230597 | Case-control study | 22 AD 18 CN | Patients were newly diagnosed of sporadic Alzheimer’s disease by the Neurologic Service of Hospital Juan Ramón Jiménez | Serum | DIMS |
| 23700429 | Case-control study | 15 AD 15 MCI 15 CN | MCSA and ADRC | Plasma ;CSF | UPLC-ToF-MS |
| 23571809 | Case-control study | 40 AD 36 MCI 38 CN | Penn Memory Center, University of Pennsylvania (Philadelphia,PA, USA) and the Maria de los Santos Health Center (Philadelphia, PA, USA), | CSF | liquid chromatography electrochemical array platform |
| 22359596 | Case-control study | 53 Light to mild AD (MMSE>22) 26 Moderate to Strong AD (MMSE 14-22) 51 CN | Five different clinical centres in Europe: One in Germany, one in France, one in Switzerland, and two in Sweden | CSF | GC-MS; LC-MS/MS |
| 23827464 | Nested case control study | 21 AD 21 MCI-S 12 MCI-AD 21 CN | Unknown | CSF | RP/UHPLC-TOF MS; HILIC/UHPLC-T OF MS |
| 20980076 | Case-control study | 46 AD 39 CN | Centre of Harbin Elderly Care Service | Serum | GC-MS |
| 22832349 | Nested case control study | 37 AD 52 Progressive MCI 91 Stable MCI 46 CN | Longitudinal study databases gathered in the University of Kuopio | Serum | UPLC-MS ; GC GC-TOFMS |
| 20457143 | Case-control study | 20 AD 20 CN | Shengjing Affiliated Hospital China Medical University. | Plasma | UPLC-MS |
| 17723614 | Case-control study | 4 AD 22 CN | Unknown | Urine | GC-SIM-MS |
| 17688973 | Case-control study | 39 AD 25 VD 24 CN | Aoisoranosato Geriatric Health Services Facility and the Arimakogen Hospital | Plasma | GC-MS |
| 14718371 | Case-control study | 3 AD 3 CN | Unknown | Brain | NMR; GC, GC-MS |
| 25575172 | Case-control study | 8 AD 21 CN | Neurological Service of Hospital Juan Ramón Jiménez | Serum | GC-MS |
| 23291240 | Case-control study | 106 AD 87 CN | Memory Clinic at the Department of Psychiatry University of Bonn | Plasma ;CSF | GC-MS and gas chromatography-fl ame ionization detection |
| 28323825 | Case-control study | 14 AD 15 ASYMAD 14 CN | BLSA | Brain | LC-MS; GC-MS |
| 27757515 | Case-control study | 6 AD 6 CN | PrecisionMed Inc. | Plasma ;CSF | UPLC-MS/MS |
| NS | Case-control study | 256 AD 218 CN | First Affiliated Hospital Heilongjiang University of Chinese Medicine. | Saliva | FUPLC-MS |
| 25192049 | Case-control study | 46 AD 47 CN | Centre of Harbin Elderly Care Service Heilongjiang Province in the North of China | Serum | UPLC-QTOF-MS |
| 29852382 | Case-control study | 10 AD 10 CN | Unknown | CSF | UHPLC-MS/MS |
| 26526912 | Case-control study | unknown | Fukushimura Brain Bank | Brain | LC-ESI-MS/MS |

**Supplementary Table 2** Mouse Metabolite Pathway Enrichment Information

| **Pathway** | **Total** | **Expected** | **Hits** | **Raw p** | **Holm p** | **FDR** | **Enrichment Ratio** |
| --- | --- | --- | --- | --- | --- | --- | --- |
| D-Glutamine and D-glutamate metabolism | 6 | 0.11 | 4 | 0.00 | 0.00 | 0.00 | 37.74 |
| Nitrogen metabolism | 6 | 0.11 | 2 | 0.00 | 0.34 | 0.05 | 18.87 |
| Arginine biosynthesis | 14 | 0.25 | 4 | 0.00 | 0.01 | 0.00 | 16.13 |
| Valine, leucine and isoleucine biosynthesis | 8 | 0.14 | 2 | 0.01 | 0.61 | 0.08 | 14.08 |
| Phenylalanine, tyrosine and tryptophan biosynthesis | 4 | 0.07 | 1 | 0.07 | 1.00 | 0.42 | 14.08 |
| Alanine, aspartate and glutamate metabolism | 28 | 0.50 | 5 | 0.00 | 0.01 | 0.00 | 10.06 |
| Purine metabolism | 65 | 1.15 | 11 | 0.00 | 0.00 | 0.00 | 9.57 |
| Phosphonate and phosphinate metabolism | 6 | 0.11 | 1 | 0.10 | 1.00 | 0.54 | 9.43 |
| Aminoacyl-tRNA biosynthesis | 48 | 0.85 | 8 | 0.00 | 0.00 | 0.00 | 9.39 |
| Histidine metabolism | 16 | 0.28 | 2 | 0.03 | 1.00 | 0.22 | 7.04 |
| Ascorbate and aldarate metabolism | 8 | 0.14 | 1 | 0.13 | 1.00 | 0.60 | 7.04 |
| Taurine and hypotaurine metabolism | 8 | 0.14 | 1 | 0.13 | 1.00 | 0.60 | 7.04 |
| Glycine, serine and threonine metabolism | 33 | 0.59 | 4 | 0.00 | 0.18 | 0.03 | 6.84 |
| Ubiquinone and other terpenoid-quinone biosynthesis | 9 | 0.16 | 1 | 0.15 | 1.00 | 0.60 | 6.25 |
| Glutathione metabolism | 28 | 0.50 | 3 | 0.01 | 0.92 | 0.11 | 6.04 |
| Pantothenate and CoA biosynthesis | 19 | 0.34 | 2 | 0.04 | 1.00 | 0.28 | 5.93 |
| Phenylalanine metabolism | 10 | 0.18 | 1 | 0.16 | 1.00 | 0.63 | 5.65 |
| Glyoxylate and dicarboxylate metabolism | 32 | 0.57 | 3 | 0.02 | 1.00 | 0.15 | 5.28 |
| Glycerophospholipid metabolism | 36 | 0.64 | 3 | 0.02 | 1.00 | 0.18 | 4.69 |
| Porphyrin and chlorophyll metabolism | 30 | 0.53 | 2 | 0.10 | 1.00 | 0.54 | 3.76 |
| Butanoate metabolism | 15 | 0.27 | 1 | 0.24 | 1.00 | 0.79 | 3.76 |
| Nicotinate and nicotinamide metabolism | 15 | 0.27 | 1 | 0.24 | 1.00 | 0.79 | 3.76 |
| Arginine and proline metabolism | 38 | 0.67 | 2 | 0.14 | 1.00 | 0.60 | 2.97 |
| Pyrimidine metabolism | 39 | 0.69 | 2 | 0.15 | 1.00 | 0.60 | 2.89 |
| Selenocompound metabolism | 20 | 0.36 | 1 | 0.30 | 1.00 | 0.94 | 2.82 |
| Ether lipid metabolism | 20 | 0.36 | 1 | 0.30 | 1.00 | 0.94 | 2.82 |
| beta-Alanine metabolism | 21 | 0.37 | 1 | 0.32 | 1.00 | 0.95 | 2.68 |
| Primary bile acid biosynthesis | 46 | 0.82 | 2 | 0.20 | 1.00 | 0.71 | 2.45 |
| Galactose metabolism | 27 | 0.48 | 1 | 0.39 | 1.00 | 1.00 | 2.09 |
| Phosphatidylinositol signaling system | 28 | 0.50 | 1 | 0.40 | 1.00 | 1.00 | 2.01 |
| Inositol phosphate metabolism | 30 | 0.53 | 1 | 0.42 | 1.00 | 1.00 | 1.88 |
| Valine, leucine and isoleucine degradation | 40 | 0.71 | 1 | 0.52 | 1.00 | 1.00 | 1.41 |
| Tyrosine metabolism | 42 | 0.75 | 1 | 0.53 | 1.00 | 1.00 | 1.34 |

**Supplementary Table 3** Human Metabolite Pathway Enrichment Information

| **Pathway** | | | | **Total** | **Expected** | **Hits** | **Raw p** | **Holm p** | **FDR** | **Enrichment Ratio** |
| --- | --- | --- | --- | --- | --- | --- | --- | --- | --- | --- |
|  |  |  | D-Glutamine and D-glutamate metabolism | 6 | 0.23 | 4.00 | 0.00 | 0.00 | 0.00 | 17.78 |
|  |  |  | Arginine biosynthesis | 14 | 0.52 | 8.00 | 0.00 | 0.00 | 0.00 | 15.27 |
|  |  |  | Neomycin, kanamycin and gentamicin biosynthesis | 2 | 0.07 | 1.00 | 0.07 | 1.00 | 0.34 | 13.35 |
|  |  |  | Phenylalanine, tyrosine and tryptophan biosynthesis | 4 | 0.15 | 2.00 | 0.01 | 0.60 | 0.07 | 13.33 |
|  |  |  | Nitrogen metabolism | 6 | 0.23 | 2.00 | 0.02 | 1.00 | 0.14 | 8.89 |
|  |  |  | Aminoacyl-tRNA biosynthesis | 48 | 1.80 | 15.00 | 0.00 | 0.00 | 0.00 | 8.33 |
|  |  |  | Histidine metabolism | 16 | 0.60 | 4.00 | 0.00 | 0.18 | 0.03 | 6.68 |
|  |  |  | Taurine and hypotaurine metabolism | 8 | 0.30 | 2.00 | 0.03 | 1.00 | 0.20 | 6.67 |
|  |  |  | Alanine, aspartate and glutamate metabolism | 28 | 1.05 | 7.00 | 0.00 | 0.00 | 0.00 | 6.67 |
|  |  |  | Linoleic acid metabolism | 5 | 0.19 | 1.00 | 0.17 | 1.00 | 0.66 | 5.35 |
|  |  |  | Phenylalanine metabolism | 10 | 0.38 | 2.00 | 0.05 | 1.00 | 0.27 | 5.33 |
|  |  |  | Biosynthesis of unsaturated fatty acids | 36 | 1.35 | 6.00 | 0.00 | 0.14 | 0.03 | 4.44 |
|  |  |  | Arginine and proline metabolism | 38 | 1.42 | 6.00 | 0.00 | 0.18 | 0.03 | 4.23 |
|  |  |  | Pantothenate and CoA biosynthesis | 19 | 0.71 | 3.00 | 0.03 | 1.00 | 0.20 | 4.21 |
|  |  |  | Glycine, serine and threonine metabolism | 33 | 1.24 | 5.00 | 0.01 | 0.51 | 0.07 | 4.03 |
|  |  |  | Thiamine metabolism | 7 | 0.26 | 1.00 | 0.24 | 1.00 | 0.78 | 3.82 |
|  |  |  | Glutathione metabolism | 28 | 1.05 | 4.00 | 0.02 | 1.00 | 0.14 | 3.81 |
|  |  |  | Pyruvate metabolism | 22 | 0.82 | 3.00 | 0.05 | 1.00 | 0.26 | 3.64 |
|  |  |  | Glyoxylate and dicarboxylate metabolism | 32 | 1.20 | 4.00 | 0.03 | 1.00 | 0.20 | 3.33 |
|  |  |  | Ascorbate and aldarate metabolism | 8 | 0.30 | 1.00 | 0.26 | 1.00 | 0.78 | 3.33 |
|  |  |  | Valine, leucine and isoleucine biosynthesis | 8 | 0.30 | 1.00 | 0.26 | 1.00 | 0.78 | 3.33 |
|  |  |  | Ubiquinone and other terpenoid-quinone biosynthesis | 9 | 0.34 | 1.00 | 0.29 | 1.00 | 0.82 | 2.97 |
|  |  |  | Citrate cycle (TCA cycle) | 20 | 0.75 | 2.00 | 0.17 | 1.00 | 0.66 | 2.67 |
|  |  |  | Tyrosine metabolism | 42 | 1.57 | 4.00 | 0.07 | 1.00 | 0.34 | 2.55 |
|  |  |  | beta-Alanine metabolism | 21 | 0.79 | 2.00 | 0.18 | 1.00 | 0.67 | 2.54 |
|  |  |  | Cysteine and methionine metabolism | 33 | 1.24 | 3.00 | 0.12 | 1.00 | 0.52 | 2.42 |
|  |  |  | Purine metabolism | 65 | 2.43 | 5.00 | 0.09 | 1.00 | 0.41 | 2.06 |
|  |  |  | Glycolysis / Gluconeogenesis | 26 | 0.97 | 2.00 | 0.25 | 1.00 | 0.78 | 2.05 |
|  |  |  | alpha-Linolenic acid metabolism | 13 | 0.49 | 1.00 | 0.39 | 1.00 | 1.00 | 2.05 |
|  |  |  | Galactose metabolism | 27 | 1.01 | 2.00 | 0.27 | 1.00 | 0.78 | 1.98 |
|  |  |  | Porphyrin and chlorophyll metabolism | 30 | 1.12 | 2.00 | 0.31 | 1.00 | 0.84 | 1.79 |
|  |  |  | Butanoate metabolism | 15 | 0.56 | 1.00 | 0.44 | 1.00 | 1.00 | 1.78 |
|  |  |  | Nicotinate and nicotinamide metabolism | 15 | 0.56 | 1.00 | 0.44 | 1.00 | 1.00 | 1.78 |
|  |  |  | Primary bile acid biosynthesis | 46 | 1.72 | 3.00 | 0.25 | 1.00 | 0.78 | 1.74 |
|  |  |  | Starch and sucrose metabolism | 18 | 0.67 | 1.00 | 0.50 | 1.00 | 1.00 | 1.48 |
|  |  |  | Pyrimidine metabolism | 39 | 1.46 | 2.00 | 0.43 | 1.00 | 1.00 | 1.37 |
|  |  |  | Selenocompound metabolism | 20 | 0.75 | 1.00 | 0.54 | 1.00 | 1.00 | 1.34 |
|  |  |  | Sphingolipid metabolism | 21 | 0.79 | 1.00 | 0.55 | 1.00 | 1.00 | 1.27 |
|  |  |  | Propanoate metabolism | 23 | 0.86 | 1.00 | 0.59 | 1.00 | 1.00 | 1.16 |
|  |  |  | Fatty acid biosynthesis | 47 | 1.76 | 2.00 | 0.53 | 1.00 | 1.00 | 1.14 |
|  |  |  | Lysine degradation | 25 | 0.94 | 1.00 | 0.62 | 1.00 | 1.00 | 1.07 |
|  |  |  | Phosphatidylinositol signaling system | 28 | 1.05 | 1.00 | 0.66 | 1.00 | 1.00 | 0.95 |
|  |  |  | Inositol phosphate metabolism | 30 | 1.12 | 1.00 | 0.69 | 1.00 | 1.00 | 0.89 |
|  |  |  | Glycerophospholipid metabolism | 36 | 1.35 | 1.00 | 0.75 | 1.00 | 1.00 | 0.74 |
|  |  |  | Fatty acid elongation | 39 | 1.46 | 1.00 | 0.78 | 1.00 | 1.00 | 0.68 |
|  |  |  | Fatty acid degradation | 39 | 1.46 | 1.00 | 0.78 | 1.00 | 1.00 | 0.68 |
|  |  |  | Valine, leucine and isoleucine degradation | 40 | 1.50 | 1.00 | 0.79 | 1.00 | 1.00 | 0.67 |
|  |  |  | Tryptophan metabolism | 41 | 1.54 | 1.00 | 0.80 | 1.00 | 1.00 | 0.65 |
|  |  |  | Steroid biosynthesis | 42 | 1.57 | 1.00 | 0.80 | 1.00 | 1.00 | 0.64 |
|  |  |  | Steroid hormone biosynthesis | 85 | 3.18 | 1.00 | 0.97 | 1.00 | 1.00 | 0.31 |

**Supplementary Table 3 Abbreviations**

| **symbol** | **Official Full Name** |
| --- | --- |
| AD | Alzheimer's disease |
| HER2 | Human EGF Receptor |
| NDF2 | Neuronal differentiation 2 |
| Aβ | β-amyloid |
| NFTs | Neurofibrillary tangles |
| FDA | Food and Drug Administration |
| PET | Positron emission tomography |
| 1H-MRS | Proton Magnetic Resonance Spectroscopy |
| PFAMs | Primary fatty acid amides |
| CSF | Cerebrospinal fluid |
| APP/PS1 | APPswe/PS1dE9 |
| 3xTg | Triple transgenic |
| 5xFAD | 5 familial AD mutations |
| MCI | Mild cognitive impairment |
| WT | Wild type |
| cAMP | Cyclic adenosine monophosphate |
| APP | Amyloid precursor protein |
| LTP | Long-term potentiation |
| GPR40 | G protein-coupled receptor 40 |
| EGFR | Epidermal growth factor receptor |
| EGF | Epithelial growth factor |

**Introduction of Mouse Animal Model**

APP/PS1 transgenic mouse models are commonly used AD models that harbor both the Swedish mutation in human amyloid precursor protein (AβPP) encoding gene and the exon-9 deletion mutation in human presenilin-1(PS1) gene (Bilkei-Gorzo and Andras, 2014). These mice generated Aβ plaques in the brain at 5~6 months old, although production of Aβ had been found as early as 3 months old. APP/PS1 mice displayed progressive age-related memory impairments, which appeared as early as 7 months old. In behavioristics testing, the mice showed significant deficiencies in spatial memory and learning ability (Bonardi et al., 2011;Xiong et al., 2011). 3xTg-AD mouse model incorporating the transgenes PS1M146V, APPSwe, and tauP301L. This model exhibits plaque and tangles pathology associated with synaptic dysfunction. Aβ deposition is progressively increased, and hyperphosphorylated tau aggregates are detectable in the hippocampus at 12~15 months of age. Tg2576 is a transgenic mouse model with the “Swedish” familial mutation of human AβPP that is characterized by a slow and progressive decline of cognitive functions as the disease progresses (Hsiao et al., 1996). Cognitive impairment in this model begins at about 6 months, and amyloid plaques begin to form at 10 months (Zhuo et al., 2008). The 5xFAD model expresses human AβPP with the Swedish (KM670/671NL), Florida (I716V), and London (V717I) mutations, together with mutant PS1 (M146L, L286V) under the control of the murine Thy-1 promotor (Oakley et al., 2006). The neuropathological changes in this model are more obvious. Aβ1-42 deposition begins at about 1.5 months old, and neuroinflammation such as microglia and astroglial hyperplasia occurs at 2 months old.

**References**

Bilkei-Gorzo, and Andras (2014). Genetic mouse models of brain ageing and Alzheimer's disease. *Pharmacology & Therapeutics* 142**,** 244-257.

Bonardi, C., Pulford, F.D., Jennings, D., and Pardon, M.C. (2011). A detailed analysis of the early context extinction deficits seen in APPswe/PS1dE9 female mice and their relevance to preclinical Alzheimer's disease. *Behavioural Brain Research* 222**,** 89-97.

Hsiao, Karen, Chapman, and Paul (1996). Correlative memory deficits, A-beta elevation, and amyloid plaques in transgenic mice. *Science*.

Oakley, H., Cole, S.L., Logan, S., Maus, E., Shao, P., Craft, J., Guillozet-Bongaarts, A., Ohno, M., Disterhoft, J., and Eldik, L.V. (2006). Intraneuronal beta-amyloid aggregates, neurodegeneration, and neuron loss in transgenic mice with five familial Alzheimer's disease mutations: potential factors in amyloid plaque formation. *Journal of Neuroscience* 26**,** 10129-10140.

Xiong, H., Callaghan, D., Wodzinska, J., Xu, J., Premyslova, M., Liu, Q.Y., Connelly, J., and Zhang, W. (2011). Biochemical and behavioral characterization of the double transgenic mouse model (APPswe/PS1dE9) of Alzheimer's disease. *Neuroscience Bulletin* 27**,** 221.

Zhuo, J.M., Prakasam, A., Murray, M., Zhang, H.Y., Baxter, M., Sambamurti, K., and Nicolle, M. (2008). An increase in Abeta42 in the prefrontal cortex is associated with a reversal-learning impairment in Alzheimer's disease model Tg2576 APPsw mice. *Current Alzheimer Research* 5**,** -.
